# Effects of Supplementing Rumen-Protected Glutathione on Lactation Performance, Nutrients, Oxidative Stress, Inflammation, and Health in Dairy Cows During the Transition Period

**DOI:** 10.3390/vetsci12020084

**Published:** 2025-01-23

**Authors:** Yu Hao, Xuejie Jiang, Rui Sun, Yunlong Bai, Chuang Xu, Yuxi Song, Cheng Xia

**Affiliations:** 1 College of Animal Science and Veterinary Medicine, Heilongjiang Bayi Agricultural University, Sartu District, Daqing 163319, China; hy0314ai@163.com (Y.H.); jxj2862109645@163.com (X.J.); a13936697304@163.com (R.S.); bai53626077@126.com (Y.B.); 2 College of Veterinary Medicine, China Agricultural University, Haidian District, Beijing 100091, China; xuchuang7175@163.com

**Keywords:** rumen-protected glutathione, lactation performance, nutrients metabolism, oxidative stress, inflammation, health

## Abstract

Glutathione (GSH), widely present in plant and animal cells and crucial for combating oxidative stress and inflammation, has not been evaluated in dairy cows. This study aims to evaluate the effects of rumen-protected glutathione (RPGSH) supplementation on lactation, nutrient metabolism, oxidative stress, inflammation, and health in transition dairy cows. Forty Holstein dairy cows (2.65 ± 0.78 of parity, 2.81 ± 0.24 of body condition score, 9207.56 ± 1139.18 kg of previous 305-day milk yield, 657.53 ± 55.52 kg of body weight, mean ± SD) were selected from a large cohort of 3215 cows on day 21 before expected calving (day −21 ± 3 d). Cows were randomly stratified into four dietary treatment groups (n = 10 per group): control (basal diet + 0 g/d RPGSH); T1 (basal diet + 1.5 g/d RPGSH); T2 (basal diet + 2 g/d RPGSH); and T3 (basal diet + 3 g/d RPGSH). Supplementation commenced approximately 21 days (±3) prepartum and continued through 21 days postpartum. Blood samples were collected at −21 ± 3, −14 ± 3, −7 ± 3, 0, 7, 14, and 21 d for analysis of serum metabolic parameters related to oxidative stress and inflammation. Milk composition was analyzed from samples collected on days 3, 7, 14, and 21 postpartum. Compared with the control group, supplementation with 2 g/d of RPGSH reduced somatic cell count (*p* < 0.05) and the incidence of postpartum diseases in dairy cows. No differences were observed among the groups in milk yield, milk fat, protein, lactose, total solids, dry matter intake, or energy-corrected milk. However, fat-corrected milk and feed efficiency were higher in the T2 group compared to the control (*p* < 0.05). Calcium and phosphorus levels did not differ among the groups. Compared to the control group, cows supplemented with 2 g/d RPGSH had lower β-hydroxybutyrate levels and higher glucose levels on days 14 and 21 postpartum (*p* < 0.05). From days 14 to 21 postpartum, RPGSH supplementation increased blood GSH, serum catalase, and total antioxidant capacity while reducing malondialdehyde, reactive oxygen species, haptoglobin, cortisol, C-reactive protein, and interleukin−6 levels compared with the control group (*p* < 0.05). The supplementation of 2 g/d RPGSH showed relatively better effects. RPGSH supplementation at 2 g/d improved lactation performance, nutrient metabolism, oxidative stress, and inflammation status in dairy cows, playing a crucial role in maintaining their health. To our knowledge, this is the first report on the effects of supplementing RPGSH additive in Holstein cows.

## Simple Summary

The transition period is a critical phase in dairy cows. This study evaluated the effects of rumen-protected glutathione supplementation on lactation, oxidative stress, inflammation, and overall health in transition dairy cows. Supplementation with 2 g/d of RPGSH was shown to reduce somatic cell count, improve fat-corrected milk yield, and enhance feed efficiency. RPGSH also increased antioxidant capacity and reduced inflammation response.

## 1. Introduction

The transition period—spanning from 21 days (d) before to 21 d after calving—is a critical phase in the lifecycle of dairy cows [1]. This period is marked by significant physiological changes predisposing cows to oxidative stress, inflammation, metabolic disorders, and diseases, adversely affecting lactation performance and health [2]. Oxidative stress exacerbates the progression and severity of various conditions by impairing cellular functions and weakening immune responses, which can lead to complications such as mastitis, endometritis, ketosis, and retained placenta [3,4]. These conditions lead to reduced milk production, higher veterinary costs, and shortened cow lifespans, resulting in significant economic losses for the dairy industry [5]. Feed additives are widely employed in transition cow diets to improve oxidative stress and inflammation, reducing disease incidences and economic losses [6,7].

Glutathione (GSH)—the most abundant intracellular thiol-containing antioxidant made of glutamate, cysteine, and glycine—protects cells from oxidative damage by scavenging free radicals [8,9]. Beyond its antioxidative role, GSH is crucial in anti-inflammatory processes, inhibiting the production and release of inflammatory mediators, especially under stress or disease conditions [10]. This dual function highlights its crucial role in regulating antioxidative defense and anti-inflammatory systems, suggesting therapeutic potential in managing oxidative stress and enhancing immune health. Dietary antioxidants supplemented during the transition period have been demonstrated to enhance dairy cow lactation performance [11,12] and improve nutrient metabolism [13]. Although GSH can be synthesized endogenously in the tissues of ruminants via the one-carbon metabolism pathway [14], the physiological challenges faced by transition dairy cows may render endogenous synthesis insufficient to meet their physiological demands. Thus, supplementation of rumen-protected glutathione (RPGSH) during this period could be of significant importance.

The specific impacts of RPGSH supplementation on lactation performance, nutrient metabolism, oxidative stress, inflammation, and health outcomes in transition cows remain underexplored. This knowledge gap emphasizes the need for targeted research to explore RPGSH’s potential as a nutritional intervention during this critical period [15,16]. Considering that all cows may experience some level of oxidative stress and inflammation postpartum [17] and that the initiation of immune responses requires substantial amounts of GSH [18,19], it is hypothesized that RPGSH supplementation could enable cows to more effectively manage oxidative stress and inflammation during the transition period, thereby potentially supporting enhanced nutrients metabolism level, lactation performance, and health status. This study evaluated the effects of RPGSH supplementation on lactation performance, nutrient metabolism, oxidative stress, inflammation, and health in dairy cows during the transition period.

## 2. Materials and Methods

### 2.1. Experimental Materials

RPGSH was provided by Beijing Feeding Feed Sci. & Tec. Co., Ltd., Beijing, China. GSH was processed using the microencapsulated per rumen technique by weighing 30.5 kg of GSH and 10 kg of water, mixing and shaping them into bar-shaped pellets using a granulator, and then rotating and boiling the pellets in a shot blasting machine, and finally drying them to a moisture content of ≤10 wt% to obtain the micro-pellets. The micro-pellets were fluidized to obtain fluidized micro-pellets. Finally, palmitic fatty acids were melted and cooled to 80 °C and encapsulated onto the surface of fluidized micro-pellets in the ratio of fatty acids to fluidized micro-pellets in the mass ratio of 68:30 to obtain granular RPGSH (30% purity). In a separate in situ trial, the rumen protection rate of RPGSH was calculated to exceed 90% based on the experimental model of Ørskov and McDonald [20] (Supplementary Table S1).

### 2.2. Experimental Design

The experiment was conducted using Holstein cows on a large intensive cattle farm in the central region of Heilongjiang Province during the winter season, spanning January to March 2024. All procedures involving cows were approved by the Animal Care and Use Committee of Heilongjiang Bayi Agricultural University (Protocol No. DWKJXY2024007). We selected 40 Holstein dairy cows from a large cohort of 3215 cows on day 21 before expected calving (day −21 ± 3 d). Cows were randomly stratified into four dietary treatment groups (n = 10 per group): control (basal diet + 0 g/d RPGSH); T1 (basal diet + 1.5 g/d RPGSH); T2 (basal diet + 2 g/d RPGSH); and T3 (basal diet + 3 g/d RPGSH). Daily, the supplement was top-dressed individually to each cow during the morning feeding when cows were locked in head stanchion lockups for a period of 30 to 45 min. Treatments began 21 ± 3 d before the expected parturition date and continued until 21 DIM. No initial differences in parity (2.65 ± 0.78), body condition score (2.81 ± 0.24), body weight (657.53 ± 55.52 kg), and 305 d milk yield (9207.56 ± 1139.18 kg) were observed among groups.

### 2.3. Feeding Management

The experimental dry cows were housed in four separate tie-stall barns, with each barn containing 10 cows from a different group. These barns were separate from the main housing facilities used for the rest of the herd. The cows were provided diet ad libitum, with a target refusal rate of 12%. Cows were milked thrice daily at 08:00, 14:00, and 22:00 by a rotary milking machine (AutoRotor PerFormer Plus, GEM Inc., Munich, Germany). Cows had access to water ad libitum, and each group was confined to a pen. Humane endpoints were established to monitor the occurrence of pain or illness daily. In our experiment, to minimize external factors that could potentially influence the results, no vaccinations or deworming treatments were administered on the farm.

### 2.4. Sample Collection and Processing

From 3 weeks prepartum to 3 weeks postpartum, the feed intake of each cow was recorded for 2 non-consecutive days per week. The dry matter intake (DMI) for each group was calculated by analyzing the dry matter content of the feed. The average of the 2 daily values recorded each week was used as the weekly DMI average for each cow. On the same day, fresh TMR and feed refusal samples were collected and analyzed for dry matter (105 °C, 4 h), crude protein [21], and neutral detergent fiber (NDF) [22]. Starch, calcium, and phosphorus contents in the feed were analyzed according to the respective China National Standard (GB/T 20194-2018), China National Standard (GB/T 6436-2018), and China National Standard (GB/T 6437-2018) [23,24,25]. The TMR formulation adhered to NRC (2001) standards [26]. Ingredients and chemical composition of the basal diet are listed in Table 1.

Milk yield was recorded on day 1 post-calving and every 3 d until 21 d postpartum. Milk samples (40 mL) from each cow were collected on 3, 7, 14, and 21 d postpartum at 08:30, 14:30, and 22:30 and thoroughly mixed in a 4:3:3 ratio. The samples were combined with bronopol (a milk preservative; D&F Control Systems Inc., San Ramon, CA, USA) and sent to a third-party laboratory for analysis using a 4-channel spectrophotometer (MilkoScan; Foss Electric A/S, Hillerød, Denmark). The analysis tested for components, including fats, proteins, lactose, urea nitrogen (UN), total solids (TS), and somatic cell count (SCC). The yield of energy-corrected milk (ECM) and fat-corrected milk (FCM) was calculated by reference to Tyrrell and Reid [28] and VanBaale et al. [29]: ECM = [0.327 × milk yield + 12.95 × fat yield + 7.2 × protein yield], and 3.5% FCM = [0.432 × milk yield + 16.23 × fat yield]. Feed efficiency was determined by the ratios of FCM to DMI.

In the morning, 10 mL of blood samples were collected from the coccygeal vein of the subjects within 10 min following feeding, on d −21 (±3 d), −14 (±3 d), −7 (±3 d), 0 (the day of calving), and 7, 14, and 21 post-calving. Subsequently, 2 mL of the whole blood was immediately dispensed into tubes containing ethylenediaminetetraacetic acid to detect GSH. The remaining 8 mL was transferred to serum separator tubes (BD Vacutainer Plus plastic serum tubes; BD Diagnostics, Franklin Lakes, NJ, USA) and centrifuged at 3500× *g* for 10 min at a temperature of 4 °C to obtain serum. The harvested serum samples were stored at −80 °C until further analysis.

Serum concentrations of calcium, phosphorus, β-hydroxybutyric acid (BHBA), and glucose were analyzed using commercial biochemical assay kits (Mindray Biomedical Electronics Co., Ltd., Shenzhen, China) by the Mindray BS-830S fully automatic biochemistry analyzer. The activities of blood GSH, serum catalase (CAT), total antioxidant capacity (T-AOC), and malondialdehyde (MDA) were determined using the Microplate Reader Multiskan FC (Thermo Fisher Scientific Co., Ltd., Shanghai, China) and commercial colorimetric kits (Nanjing Jiancheng Bioengineering Institute Co., Ltd., Nanjing, China). Serum levels of reactive oxygen species (ROS), haptoglobin (HP), C-reactive protein (CRP), cortisol (COR), and interleukin-6 (IL-6) were measured using commercially available bovine-specific enzyme-linked immunosorbent assay kits (Shanghai Lengton Bioscience Co., Ltd., Shanghai, China). All measurements were performed following the manufacturer’s instructions.

### 2.5. Data Collection

Age, parity, milk yield, and incidences of postpartum diseases (ketosis, mastitis, and metritis) were recorded using the specific software (version 5.5, Afifarm, Afimilk, Kibbutz Afikim, 1514800, Israel). Two trained farm veterinarians evaluated the body condition score weekly using the established five-point method [30]. Clinical ketosis was diagnosed based on blood BHBA ≥ 3.0 mmol/L; subclinical ketosis was diagnosed based on blood BHBA > 1.2 mmol/L [31]. Mastitis was identified based on the SCC (>200,000/mL) in milk [32]. Metritis in cows was characterized by evaluating the visual appearance (including color, consistency, and presence of pus) and odor (either absent or foul) of vaginal discharge. Cows with malodorous discharge were typically diagnosed with metritis, often scoring between 2 and 4 on diagnostic scales [33]. Additionally, throughout the experimental period, no postpartum diseases such as abomasum displacement or hypocalcemia were observed in the cows.

### 2.6. Statistical Analysis

The Kolmogorov–Smirnov test was employed to assess normal distribution, while Levene’s test was utilized to examine the homogeneity of variances. Tukey’s honest significance Difference (HSD) post hoc test was used to identify specific inter-group differences while controlling for multiple comparisons. The statistical analysis was conducted using the Statistical Package for Social Sciences software (version 26.0; IBM, Armonk, NY, USA). The GraphaPad Prism software (version 9) was used to create graphs. DMI, milk yield, milk composition, and biochemical indicators were examined using a mixed model procedure to accommodate correlated repeated measurements. The fixed effects in the model included treatment (T1, T2, T3, and control), time, and the interaction between them; the model is as follows:

$$Y_{\mathrm{ijk}}=\mu +{\mathrm{trt}}_{i}+{\mathrm{time}}_{j}+{\left(\mathrm{trt}\;\times \;\mathrm{time}\right)}_{\mathrm{ij}}+{\mathrm{cow}}_{k}+e_{\mathrm{ijk}}$$

where Y_ijk_ is a measure of the dependent variable of the j-th cow of the i-th treatment on the k-th time; μ is the overall mean; trt_i_ is the fixed treatment effect; time_j_ is the fixed time effect; (trt × time)_ij_ is the interaction between treatment and time; cow_k_ is the random residual effect; e_ijk_ is is the random residual effect. To evaluate the effects of increasing RPGSH supplementation on the measured parameters, both linear and quadratic relationships were analyzed.

Pearson’s correlation coefficient was applied to evaluate the correlation between blood GSH concentration and serum biochemical indicators. Descriptive statistics were used to characterize the incidences of postpartum diseases. Spearman rank correlation coefficient was used to test the correlation between blood GSH level and postpartum diseases. Statistical significance was determined based on the probability (*p*) values, with values < 0.05 considered significant.

## 3. Results

### 3.1. DMI, Milk Yield, and Milk Composition

The results for DMI, milk yield, and milk composition are presented in Table 2. No differences among the dietary groups in DMI, milk yield, milk fat, protein, lactose, UN, TS, and ECM (*p* > 0.05). A quadratic decrease in SCC was observed with increasing levels of RPGSH supplementation (Quad *p* = 0.049), while FCM (Quad *p* = 0.032) and feed efficiency (Quad *p* = 0.044) had quadratic increases at higher levels of RPGSH supplementation. Significant changes were observed over time for milk yield, DMI, milk components (except milk fat), ECM, FCM, and feed efficiency (*p* < 0.05). The interaction between treatment and time for all parameters were not significant (*p* > 0.05).

### 3.2. Effects of RPGSH Supplementation on Serum Metabolites in Dairy Cows

As revealed in Table 3, no differences were observed in calcium and phosphorus (*p* > 0.05) among the groups. Compared to the control group, the T2 group exhibited lower BHBA concentration (*p* < 0.05) and higher glucose concentration (*p* < 0.05) at 14 and 21 d postpartum. Serum BHBA and glucose levels changed significantly over time (*p* < 0.05), while serum calcium and phosphorus levels did not change. No significant interactions between treatment and time were found for calcium, phosphorus, BHBA, and glucose levels (*p* > 0.05).

### 3.3. Effects of RPGSH Supplementation on Oxidative Stress Biomarkers in Dairy Cows

**Figure 1:**
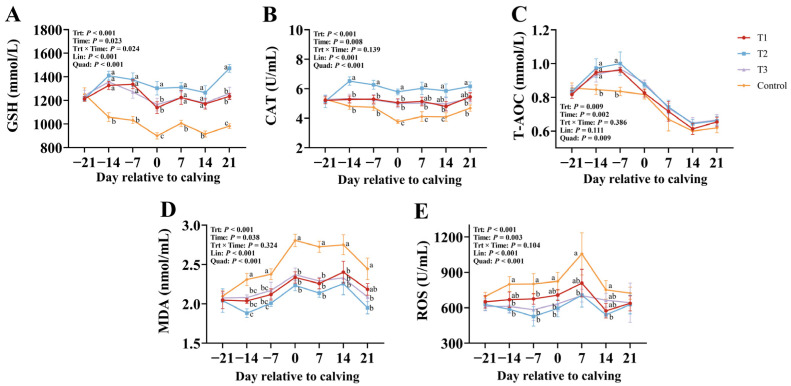
Effects of rumen-protected glutathione (RPGSH) on glutathione (GSH, (**A**)), catalase (CAT, (**B**)), total antioxidant capacity (T-AOC, (**C**)), malondialdehyde (MDA, (**D**)), and reactive oxygen species (ROS, (**E**)). Treatment: T1 = basal diet + RPGSH 1.5 g/d (shown as ●), T2 = basal diet + RPGSH 2 g/d (shown as ■), T3 = basal diet + RPGSH 3 g/d (shown as ▲), and control = basal diet (shown as ⯁); Lin = linear; Quad = quadratic. Error bars indicate the SEM. Different lowercase letters indicate significant differences between peers (*p* < 0.05).

Figure 1 demonstrates the effects of dietary RPGSH supplementation on oxidative stress biomarkers. Compared to the control group, RPGSH supplementation significantly increased blood GSH levels, as well as serum CAT and T-AOC levels (*p* < 0.05), while decreasing serum MDA and ROS levels (*p* < 0.05), particularly at a dosage of 2 g/d, where blood GSH levels and serum CAT and T-AOC levels were the highest, and serum MDA and ROS levels were the lowest. Moreover, a temporal effect was observed, with blood GSH and serum CAT, T-AOC, MDA, and ROS levels showing significant changes over the experimental period (*p* < 0.05). The blood GSH concentration in the RPGSH groups increased significantly during the period from −21 d to −14 d, while the blood GSH concentration in the control group decreased significantly during the same period, resulting in a significant interaction effect between treatment and time (*p* = 0.024).

### 3.4. Effects of RPGSH Supplementation on Inflammation Biomarkers in Dairy Cows

**Figure 2:**
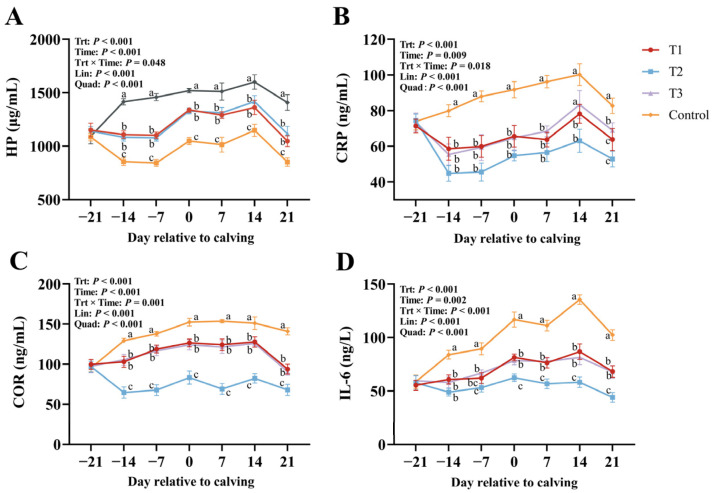
Effects of rumen-protected glutathione (RPGSH) on haptoglobin (HP, (**A**)), cortisol (COR, (**B**)), C-reactive protein (CRP, (**C**)), and interleukin-6 (IL-6, (**D**)). Treatments: T1 = basal diet + RPGSH 1.5 g/d (shown as ●), T2 = basal diet + RPGSH 2 g/d (shown as ■), T3 = basal diet + RPGSH 3 g/d (shown as ▲), and control = basal diet (shown as ⯁); Lin = linear; Quad = quadratic. Error bars indicate the SEM. Different lowercase letters indicate significant differences between peers (*p* < 0.05).

Figure 2 demonstrates the effect of supplementing RPGSH on inflammatory factors in bovine blood. With increasing RPGSH supplementation levels, serum concentrations of HP, CRP, COR, and IL-6 showed significant linear and quadratic decreases (Lin *p* < 0.001; Quad *p* < 0.001). The T2 group demonstrated relatively better-pronounced effects. All parameters were significantly affected by time (*p* < 0.05). In the RPGSH groups, HP and CRP concentrations decreased significantly from −21 d to −7 d, whereas the control group showed significant increases in HP and CRP concentrations during the same period, resulting in significant interactions between treatment and time (*p* < 0.05). Additionally, serum COR and IL-6 levels in the RPGSH groups decreased significantly during the period from −21 d to −14 d prepartum, while the control group exhibited significant increases in serum COR and IL-6 levels during the same period, further leading to significant interaction between treatment and time (*p* < 0.001).

### 3.5. Correlation Between Blood GSH Concentration and Postpartum Serum Indices

The results of Pearson correlation analysis between blood GSH concentration and postpartum serum indices in dairy cows are summarized in Table 4. No correlation was observed between blood GSH concentration and serum levels of calcium, phosphorus, BHBA, and glucose throughout the transition period (*p* > 0.05). But, blood GSH levels were significantly negatively correlated with BHBA level 14 d postpartum and significantly positively correlated with blood glucose level 14 d postpartum (*p* < 0.05; data are not displayed). On the day of calving and after calving, blood GSH level was significantly positively correlated with the level of T-AOC (*p* < 0.05). It was significantly positively correlated with the T-AOC (*p* < 0.001) level before calving. Throughout the transition period, blood GSH levels showed a highly significant positive correlation with CAT level (*p* < 0.05) but a significant negative correlation with MDA, ROS, HP, COR, CRP, and IL-6 levels (*p* < 0.001).

### 3.6. Incidence of Postpartum Diseases and Correlation with Blood GSH Levels in Dairy Cows

Table 5 describes the incidences of postpartum diseases in dairy cows. Cows in the RPGSH groups experienced fewer health issues compared to those in the control group, with the T2 group having the fewest health events. The correlation between blood GSH levels and postpartum diseases in dairy cows was analyzed using Spearman’s rank correlation test (Table 6). Prepartum blood GSH levels were significantly negatively correlated with ketosis (*p* < 0.05) and highly significantly negatively correlated with mastitis and metritis (*p* < 0.01). On the day of calving, blood GSH levels showed a highly significant negative correlation with ketosis, mastitis, and metritis (*p* < 0.01). Postpartum blood GSH levels were significantly negatively correlated with ketosis and mastitis (*p* < 0.05) and highly significantly negatively correlated with metritis (*p* < 0.01).

## 4. Discussion

This study investigated the effects of RPGSH on transition dairy cows regarding lactation performance, nutrient metabolism, oxidative stress, inflammatory factors, and health. Incorporating RPGSH into the diet enhanced the antioxidative and anti-inflammatory abilities of dairy cows during the transition period. Specifically, dietary supplementation with 2 g/d of RPGSH demonstrated the potential to enhance milk quality parameters while reducing disease incidences.

Dietary supplementation with 2 g/d of RPGSH resulted in an 8.66% increase in milk yield. Moreover, RPGSH supplementation significantly enhanced FCM production and feed efficiency without affecting DMI. These findings underscore the potential of RPGSH to enhance lactation performance in dairy cows during the transition period. This is consistent with the findings of previous studies in that supplementing antioxidants can improve the oxidative status of cows, thereby indirectly influencing their lactation performance [12,13,34]. Notably, while RPGSH supplementation increased milk yield, its effects on milk fat percentage, protein percentage, UN, and TS were insignificant, but SCC was significantly decreased. This suggests that RPGSH primarily influences lactation performance by enhancing energy balance and mitigating oxidative stress rather than directly modifying milk components. Concurrently, Santos et al. [35] proposed that specific nutritional interventions, including fatty acid supplementation, could directly impact milk fat synthesis pathways, significantly affecting milk fat content. This discrepancy reflects the potential mechanistic differences between various nutritional interventions (for instance, antioxidants vs. specific nutrients) in regulating lactation performance. The results of this study emphasize the need for future nutritional management strategies to consider the comprehensive impact of intervention measures on all aspects of dairy cow lactation performance, including milk quantity and composition [36]. Moreover, transitional nutritional management significantly impacts subsequent lactation performance. The rational nutritional interventions not only increase short-term milk yield but also enhance overall health status, reduce the incidences of metabolic disorders, and establish a robust foundation for sustained lactation performance [15,17,31]. Therefore, while the supplementation strategy with RPGSH primarily observed an increase in milk yield during the experimental period, its potential effects on improving the overall health status and long-term lactation performance of dairy cows warrant further exploration. In summary, compared to the findings of other studies, this study highlighted the potential and mechanisms of RPGSH in improving lactation performance during the transition period for dairy cows. Future research should further investigate the effects of different nutritional intervention strategies on milk composition adjustment and long-term lactation performance to promote more refined nutritional management practices.

Transitional cows, confronted with reproductive challenges, are susceptible to nutrient imbalances, which consequently diminish productivity and predispose them to diseases such as ketosis and mastitis, thereby inflicting considerable economic losses on the dairy sector [37,38,39]. In this study, 2 g/d of RPGSH supplementation significantly improved the nutrient metabolism status of dairy cows without affecting calcium and phosphorus levels, as evidenced by lower postpartum levels of BHBA and higher blood glucose levels. This indicates that RPGSH supplementation could improve the nutrient state during the transition period, aligning with findings from Meikle et al. [1], who observed that antioxidant supplementation effectively improved animal nutrient metabolism, reducing the risk of metabolic diseases caused by negative energy balance. Anyway, according to Musco et al. (2020), the level of NEFAs in each group falls in the physiological range for the first stage of lactation [40]. Conversely, some researchers revealed that while specific nutritional intervention measures may improve blood biochemical parameters, their direct enhancement effect on actual milk yield performance is not always evident [15,41,42]. This discrepancy may arise from differences in supplement types, dosages, and supplementation strategies [43,44]. Therefore, this study emphasizes the potential of a specific type of antioxidant—RPGSH—to improve the nutrient status of transitional dairy cows, providing a new perspective and strategy for their nutritional management.

During the transition period, dairy cows undergo significant physiological changes, including fetal growth during pregnancy, calving, and postpartum recovery [45,46,47]. These physiological changes may increase oxidative stress and inflammation within dairy cows, thereby increasing their susceptibility to diseases and reducing their production and reproductive performance [48,49]. In this study, the RPGSH supplementation significantly increased blood GSH, serum CAT, and T-AOC levels while significantly reducing serum levels of ROS, MDA, HP, COR, CRP, and IL-6, indicating an effective mitigation of oxidative stress and inflammation. These findings are consistent with the observations of Nikkhah and Alimirzaei [2], who found that nutritional intervention to increase antioxidant levels could effectively reduce oxidative stress and inflammation in dairy cows during the transition period, thereby improving their health status and production performance. Additionally, research by Olthof et al. [5] emphasized the importance of alleviating oxidative stress and inflammation in enhancing the production performance of dairy cows. However, while alleviating oxidative stress and inflammation is crucial for improving the overall health and production performance of dairy cows, the types of antioxidants and supplementation schemes used in different studies vary greatly, leading to inconsistencies in results. The RPGSH used in this study, as a special antioxidant with unique rumen-protective properties, may effectively function within the cows, possibly accounting for the discrepancies between this study’s results and those of other research. This study’s unique and meaningful results have been confirmed through in-depth comparative analysis, highlighting the need for future research to explore the specific impacts of different types and dosages of nutritional interventions on the health and production performance of transitional dairy cows. This is essential for optimizing the nutritional management strategies for transitional dairy cows.

In this study, RPGSH supplementation reduced the incidences of postpartum diseases, including mastitis, ketosis, and metritis, in transition period dairy cows. Particularly in the T2 group (supplemented with 2 g/d RPGSH), the overall incidence rate of diseases was significantly lower compared to the control group. Concurrent analyses of blood GSH concentration and diseases revealed significant negative correlations between blood GSH levels and the incidences of ketosis, mastitis, and metritis. This suggests that RPGSH supplementation could reduce the risk of common diseases during the transition period by improving the nutrient metabolism, oxidative stress, and inflammation status of dairy cows, thereby decreasing economic losses in the dairy industry. A study by Abuelo et al. [45] indicated that the oxidative stress status of transition-period dairy cows is closely linked to their health status, especially in direct correlation with diseases, including mastitis and ketosis. They proposed that nutritional interventions to improve the oxidative stress status of dairy cows could effectively reduce the incidences of these diseases. Additionally, Drackley [15] emphasized the importance of optimizing the nutritional status of transition-period dairy cows in preventing postpartum diseases. Although their research mainly focused on different types of nutritional interventions, this study utilized a specific antioxidant, RPGSH, as supplementation, thereby providing a new strategy for reducing the occurrence of transition period diseases. While this study emphasizes the potential of RPGSH in reducing transition period diseases, the specific mechanisms still require further investigation.

## 5. Conclusions

Feeding RPGSH during the transition period affected the nutrient metabolism, oxidative stress, and inflammation status of dairy cows, as indicated by higher glucose content and lower BHBA content from 14 to 21 d postpartum. Additionally, cows in the 2 g/d RPGSH group exhibited higher blood GSH and serum CAT and T-AOC concentrations but lower serum MDA, ROS, HP, CRP, COR, and IL-6 concentrations from d −14 to 21 relative to calving. Notably, higher RPGSH dosages did not provide additional benefits in this study. The RPGSH supplementation may have a higher dosage-independent beneficial effect in supporting the physiologic adaptations after calving, resulting in higher FCM, feed efficiency, and lower milk SCC, incidences of ketosis, mastitis, and metritis. This is the first report on the effects of supplementing RPGSH additive in Holstein cows. Further research is warranted to examine how feeding RPGSH affects microbial populations and alters the rumen fermentation patterns of dairy cows during the transition period.

## Figures and Tables

**Table 1:** Composition and nutrient level of the basal diet.

Item	Prepartum	Postpartum
Ingredients (% of DM)		
Soybean meal	2.66	4.31
DDGS	3.72	-
Alfalfa (first-cut hay)	2.21	5.74
Sugar beet pulp	8.86	2.87
Corn gluten meal	4.43	1.72
Oat grass	11.52	2.30
Corn	8.86	17.51
Silage	39.86	34.45
CaCO_3_	0.89	-
H_2_O	13.29	11.48
Na_2_CO_3_	-	0.71
CaHPO_4_	-	0.29
Fat powder	-	0.86
Rumen-protected glucose	-	1.44
Cottonseed	-	2.87
Urea	-	0.52
Soybean husk	-	7.18
Molasses	-	2.87
Premix ^1^	3.70	2.88
Nutrient level, % of DM		
Crude protein	15.7	16.7
Starch	16.8	21.5
NE_L_^2^, MCal/kg	1.52	1.76
NDF	33.7	30.7
Calcium	1.00	1.00
Phosphorus	0.40	0.40

DM = dry matter; DDGS = distillers dried grain with soluble; NDF = neutral detergent fiber. ^1^ Concentration per kilogram of premix DM: 40,000 IU of vitamin A, 37,000 IU of vitamin D, 500 IU of vitamin E, 30 mg of copper, 25 mg of iron, 140 mg of manganese, 140 mg of zinc, and 0.8 mg of selenium. ^2^ Net energy for lactation, calculated based on the Ministry of Agriculture of China recommendations [27].

**Table 2:** Effects of rumen-protected glutathione (RPGSH) on milk yield, DMI, and milk composition in transition dairy cows.

Item ^1^	Treatment ^2^				SEM	*p*-Value				
	T1	T2	T3	Control		Trt	Time	Trt × Time	Lin	Quad
Milk yield, kg/d	31.28	33.00	31.09	30.37	0.946	0.495	<0.001	0.980	0.428	0.185
DMI ^3^, kg/d	16.17	16.45	16.34	16.15	0.170	0.798	<0.001	0.711	0.833	0.315
Fat, %	3.83	4.06	3.83	3.74	0.053	0.699	0.164	0.840	0.600	0.412
Protein, %	3.43	3.42	3.38	3.45	0.051	0.943	0.028	0.704	0.995	0.651
Lactose, %	4.81	4.81	4.84	4.80	0.011	0.597	<0.001	0.217	0.734	0.524
SCC, cells/μL	121.93	101.68 ^a^	116.70	138.25 ^b^	8.07	0.140	0.083	0.964	0.217	0.049
UN, mg/dL	13.41	13.80	13.49	14.21	0.389	0.845	<0.001	0.851	0.482	0.849
TS, %	13.51	13.26	13.29	13.14	0.075	0.570	<0.001	0.412	0.612	0.236
ECM, kg/d	32.58	35.62	32.30	30.76	1.14	0.307	0.005	0.975	0.234	0.051
FCM, kg/d	35.54	39.12 ^a^	35.43	33.06 ^b^	1.17	0.235	0.008	0.907	0.167	0.032
Feed efficiency ^4^	1.92	2.06 ^a^	1.89	1.76 ^b^	0.075	0.306	0.003	0.984	0.169	0.044

DMI = Dry matter intake; SCC = Somatic cell count; UN = Urea nitrogen; ECM = Energy-corrected milk; FCM = Fat-corrected milk; TS = Total solids; Trt = treatment; Lin = linear; Quad = quadratic. ^1^ Milk samples were collected at 3, 7, 14, and 21 days postpartum. ^2^ T1 = basal diet + RPGSH 1.5 g/d, T2 = basal diet + RPGSH 2 g/d, T3 = basal diet + RPGSH 3 g/d, and control = basal diet. ^3^ DMI was recorded from three weeks before calving to three weeks after calving; the weekly average was calculated based on data collected on two non-consecutive days each week. ^4^ Efficiency of milk yield (kg of milk yield/kg of DMI). Different lowercase letters indicate significant differences between peers (*p* < 0.05).

**Table 3:** Effects of rumen-protected glutathione (RPGSH) on serum nutrients of dairy cows.

Item	Time (d)	Treatment ^1^				SEM	*p*-Value				
		T1	T2	T3	Control		Trt	Time	Trt × Time	Lin	Quad
Calcium, mmol/L	−21	2.21	2.14	2.23	2.28	0.013	0.427	0.150	0.504	0.298	0.937
	−14	2.27	2.34	2.30	2.41						
	−7	2.16	2.26	2.23	2.34						
	0	2.25	2.32	2.27	2.33						
	7	2.11	2.30	2.18	2.23						
	14	2.20	2.19	2.13	2.09						
	21	2.25	2.21	2.23	2.17						
Phosphorus, mmol/L	−21	2.00	1.94	1.95	1.97	0.021	0.114	0.21	0.940	0.821	0.103
	−14	2.09	1.99	2.07	2.03						
	−7	2.04	1.98	1.96	1.89						
	0	1.97	1.98	2.00	1.96						
	7	2.18	2.12	2.08	2.11						
	14	1.99	1.97	2.00	2.02						
	21	2.12	1.99	2.03	2.01						
BHBA, mmol/L	−21	0.64	0.49	0.55	0.73	0.017	0.169	<0.001	0.366	0.247	0.108
	−14	0.64	0.72	0.78	0.80						
	−7	0.56	0.61	0.61	0.65						
	0	0.69	0.65	0.66	0.73						
	7	0.78	0.64	0.55	0.62						
	14	0.93	0.75 ^a^	0.93	0.93 ^b^						
	21	0.82	0.68 ^a^	0.86	0.91 ^b^						
Glucose, mmol/L	−21	3.62	3.67	3.62	3.60	0.032	0.183	<0.001	0.878	0.072	0.035
	−14	3.33	3.43	3.42	3.37						
	−7	3.97	3.83	3.85	3.85						
	0	4.40	4.52	4.50	4.38						
	7	3.35	3.67	3.48	3.31						
	14	3.18	3.40 ^a^	3.15	2.97 ^b^						
	21	3.28	3.47 ^a^	3.27	2.85 ^b^						

BHBA = β-hydroxybutyric acid; Trt = treatment; Lin = linear; Quad = quadratic. ^1^ T1 = basal diet + RPGSH 1.5 g/d, T2 = basal diet + RPGSH 2 g/d, T3 = basal diet + RPGSH 3 g/d, and control = basal diet. Different lowercase letters indicate significant differences between peers (*p* < 0.05).

**Table 4:** Correlation analysis between blood GSH concentration and postpartum hematological indices in dairy cows.

Item ^1^	Prepartum ^2^		Day of Calving ^3^		Postpartum ^4^	
	*R*-Value	*p*-Value	*R*-Value	*p*-Value	*R*-Value	*p*-Value
CAT	0.820 **	<0.001	0.767 **	<0.001	0.712 **	<0.001
T-AOC	0.467 **	0.002	0.348 *	0.028	0.341 *	0.015
MDA	−0.773 **	<0.001	−0.693 **	<0.001	−0.829 **	<0.001
ROS	−0.473 **	0.002	−0.454 **	0.001	−0.462 **	<0.001
HP	−0.821 **	<0.001	−0.802 **	<0.001	−0.849 **	<0.001
COR	−0.778 **	<0.001	−0.801 **	<0.001	−0.850 **	<0.001
CRP	−0.794 **	<0.001	−0.704 **	<0.001	−0.733 **	<0.001
IL−6	−0.838 **	<0.001	−0.756 **	<0.001	−0.859 **	<0.001

BHBA = β-hydroxybutyric acid; CAT = catalase; T-AOC = total antioxidant capacity; MDA = malondialdehyde; ROS = reactive oxygen species; HP = haptoglobin; COR = cortisol; CRP = C-reactive protein; IL-6 = interleukin-6. ^1^ The mean serum indexes of dairy cows at three time points: 7; 14; and 21 d. ^2^ The mean GSH level in the blood of each cow at three time points: −21, −14, and −7 d. ^3^ The blood GSH level of each cow on the day of calving. ^4^ The mean GSH level in the blood of each cow at three time points: 7, 14, and 21 d. *R*-value indicates Pearson’s correlation coefficient, *R* > 0 indicates positive correlation, and *R* < 0 indicates negative correlation. * indicates significance at the 0.05 level, and ** indicates significance at the 0.01 level.

**Table 5:** Descriptive statistics for the incidences (%) of postpartum diseases in five groups of dairy cows.

Item	Treatment ^1^			
	T1	T2	T3	Control
*N*	10	10	10	10
Ketosis	10 (1/10)	-	10 (1/10)	1 (1/10)
Mastitis	10 (1/10)	-	10 (1/10)	2 (2/10)
Metritis	10 (1/10)	10 (1/10)	10 (1/10)	3 (3/10)

^1^ T1 = basal diet + RPGSH 1.5 g/d; T2 = basal diet + RPGSH 2 g/d; T3 = basal diet + RPGSH 3 g/d; Control = basal diet. The disease conditions of cows in each group were as follows: T1 group (n = 2): 1 cow with ketosis; 1 cow with concurrent mastitis and metritis; T2 group (n = 1): 1 cow with metritis; T3 group (n = 2): 1 cow with ketosis, 1 cow with concurrent mastitis and metritis, 1 cow with metritis; Control group (n = 3): 1 cow with concurrent ketosis and metritis, 2 cows with concurrent mastitis and metritis.

**Table 6:** Correlation analysis between blood GSH level with postpartum diseases in dairy cows.

Disease	Prepartum ^1^		Day of Calving ^2^		Postpartum ^3^	
	*R*-Value	*p*-Value	*R*-Value	*p*-Value	*R*-Value	*p*-Value
Ketosis	−0.308 *	0.029	−0.374 **	0.007	−0.336 *	0.017
Mastitis	−0. 448 **	0.001	−0.488 **	<0.001	−0.357 *	0.011
Metritis	−0.462 **	0.001	−0.428 **	0.002	−0.365 **	0.009

^1^ The mean GSH level in the blood of each cow at three time points: −21; −14; and −7 d. ^2^ The blood GSH level of each cow on the day of calving. ^3^ The mean GSH level in the blood of each cow at three time points: 7; 14; and 21 d. *R*-value indicates Spearman’s rank correlation coefficient, and *R* < 0 indicates a negative correlation. * indicates significance at the 0.05 level, and ** indicates significance at the 0.01 level.

## Data Availability

All data for this study are available from the corresponding authors.

## Supplementary Materials

The following supporting information can be downloaded at https://www.mdpi.com/article/10.3390/vetsci12020084/s1, Table S1: Ruminal degradation rate and degradation parameters of RPGSH.
