# Supplementary material for: Effects of Supplementing Rumen-Protected Glutathione on Lactation Performance, Nutrients, Oxidative Stress, Inflammation, and Health in Dairy Cows During the Transition Period
Source: Vet Sci. 2025 Jan 23;12(2):84. doi: 10.3390/vetsci12020084 (PMC11860859; doi:10.3390/vetsci12020084)
Supplement: Supplementary file 1 [file vetsci-12-00084-s001.zip › vetsci-3413172-supplementary.pdf]

Effects of supplementing rumen-protected glutathione on lactation performance, nutrients, oxidative stress, inflammation, and health in dairy cows during the transition period

Yu Hao [1,†], Xuejie Jiang [1,†], Rui Sun [1], Yunlong Bai [1], Chuang Xu [2], Yuxi Song [1,*] and Cheng Xia [1,*]

1 College of Animal Science and Veterinary Medicine, Heilongjiang Bayi Agricultural University,
Sartu District, Daqing 163319, China; hy0314ai@163.com (Y.H.); jxj2862109645@163.com (X.J.); a13936697304@163.com (R.S.); bai53626077@126.com (Y.B.)

2 College of Veterinary Medicine, China Agricultural University, Haidian District, Beijing 100091, China;
xuchuang7175@163.com

* Correspondence: syxalz@163.com (Y.S.); xcwlxyf2014@163.com (C.X.)

† These authors contributed equally to this work.

MATERIALS AND METHODS

Animals

The degradation trial involved six health Holstein cows (3.83 ± 0.17 years of age, 2.67 ± 0.21 of parity, 3.17 ± 0.14 of BCS, mean ± SEM) with permanent rumen cannulas. Cows were fed three times daily. The components of the TMR diet included soybean hulls 1.50 kg, oat grass 0.50 kg, cottonseed 1.03 kg, alfalfa 2.50 kg, soybean meal 1.30 kg, pressed corn 2.00 kg, molasses 1.00 kg, silage 25.37 kg, corn 3.00 kg, high-yield concentrate 4.09 kg. Feed analysis showed 48.00% of dry matter, 17.70% of crude protein, 7.322 MJ kg^{-1} net lactation production, 22.70% of starch, 31.50% of neutral detergent fiber, 19.00% of acid detergent fiber, 180 g of calcium, and 116 g of phosphorus.

The ruminal degradation of rumen-protected glutathione (RPGSH) was

determined using the semi-in situ nylon bag technique. First, 5 g of accurately weighed RPGSH sample was sealed in a nylon bag (8.0 cm × 12.0 cm, pore size 35-50 μm). The prepared nylon bags were dried to a constant weight at 40°C in an oven and weighed. The bags were then introduced into the rumen ventral sac via the fistula prior to morning feeding. Bags were retrieved at 0, 2, 4, 8, 12, and 24 h post-incubation. After retrieval, the bags were gently rinsed in running water for 5-10 min until the water was clear. The 0 h control group was also rinsed in water to standardize the washing procedure.For each cow, two replicates of nylon bags were used per time point. Following rinsing, the nylon bags were dried to a constant weight at 40°C in an oven and weighed again. The ruminal degradation rate of RPGSH was calculated as follows: ruminal degradation rate of RPGSH = (Weight of sample in the bag Weight of sample in the bag – Weight after ruminal digestion) × 100%. Ruminal degradation parameters and effective degradability (ED) were estimated based on the exponential model of Ørskov and McDonald (1979):

$$P = \mathrm{a} + \mathrm{b}(1 - \mathrm{e}^{-\mathrm{ct}})$$

$$ED = \mathrm{a} + \mathrm{bc}/(\mathrm{c} + \mathrm{k})$$

In the equations, *t* represents the retention time of the feedstuff in the rumen (h); *P* is the ruminal degradation rate of RPGSH at time *t* (%); a is the rapidly degradable fraction of the nutrient (%); *b* is the slowly degradable fraction of the nutrient (%); and c is the degradation rate of *b* (%/h). *ED* is the effective degradability (%) and *k* is the passage rate of the feedstuff, set at a value of 0.03.

Statistical Analysis

The data was initially summarized using Excel 2019 and analyzed with SPSS 26.0 software. The results are expressed as mean ± SD.

RESULTS

Supplementary Table S1 Ruminal degradation rate and degradation parameters of RPGSH.

Items	RPGSH
Ruminal degradability	
0 h	1.47 ± 0.20
2 h	3.16 ± 0.17
4 h	4.40 ± 0.22
8 h	6.45 ± 0.23
12 h	8.42 ± 0.36
24 h	10.55 ± 0.44
Ruminal degradation parameters	
a/%	1.45 ± 0.17
b/%	10.55 ± 0.61
c/%	0.09 ± 0.01
a + *b*/%	11.08 ± 3.38
ED/%	9.24 ± 0.40

"*a*" is the rapidly degradable fraction of the nutrient (%); "*b*" is the slowly degradable fraction of the nutrient (%); and "*c*" is the degradation rate of "*b*" (%/h). "*ED*" is the effective degradability (%).
